# Successful Management of a Pancreatic Abscess in a Dog with Juvenile Diabetes Mellitus Through Ultrasound-Guided Drainage and Medical Therapy

**DOI:** 10.3390/vetsci12070604

**Published:** 2025-06-20

**Authors:** Alexandra Daravigka, Stefanos Ninis, Panagiotis Bourdekas, Alexandros O. Konstantinidis, Argyrios Ginoudis, Katerina K. Adamama-Moraitou, Maria Lyraki, Nektarios Soubasis

**Affiliations:** 1 Companion Animal Clinic (Medicine Unit), School of Veterinary Medicine, Faculty of Health Sciences, Aristotle University of Thessaloniki, 54627 Thessaloniki, Greece; stevenninis@gmail.com (S.N.); alexkon@vet.auth.gr (A.O.K.); kadamama@vet.auth.gr (K.K.A.-M.); nsouba@vet.auth.gr (N.S.); 2 Private Practice, 54454 Thessaloniki, Greece; bourdekas_p@hotmail.com; 3 Diagnostic Laboratory, School of Veterinary Medicine, Faculty of Health Sciences, Aristotle University of Thessaloniki, 54627 Thessaloniki, Greece; agkinou@vet.auth.gr; 4 Plakentia Veterinary Clinic, 15343 Athens, Greece; m.lyraki@plakentiavet.gr

**Keywords:** pancreas, abscess, conservative treatment, diabetes mellitus, canine, minimally invasive, cytology

## Abstract

A five-month-old female mixed-breed dog presented with a two-week history of polyuria, polydipsia, and vomiting. Clinical examination revealed poor body condition, growth retardation, pale oral mucous membranes, weak pulse, and prolonged capillary refill time. Laboratory findings included neutrophilic leukocytosis with a regenerative left shift, fasting hyperglycemia, elevated fructosamine, glycated hemoglobin, and β-hydroxybutyrate concentrations, while the acid–base balance remained normal. Canine-specific pancreatic lipase and trypsin-like immunoreactivity concentrations ruled out an underlying pancreatitis or exocrine pancreatic insufficiency, respectively. Urinalysis showed glycosuria and ketonuria. Supportive care included antibiotics and regular insulin administration. Abdominal ultrasonography identified a pancreatic cavity with a thick wall and mixed echogenic fluid. Ultrasound-guided drainage was performed without complications. Cytology confirmed a pancreatic abscess with pyogranulomatous inflammation, though the culture results were negative. The dog was discharged with intermediate-acting lente insulin. Follow-up ultrasonographic evaluations at 7, 14, and 21 days and 5 months post-drainage showed no recurrence. The diabetes remained well-controlled one year post-discharge. This case report describes the successful management of a dog with juvenile diabetes mellitus complicated by a pancreatic abscess, highlighting the effectiveness of percutaneous ultrasound-guided drainage combined with medical therapy.

## Simple Summary

Pancreatic abscesses are uncommon in veterinary medicine and typically result from complications of acute pancreatitis. They are defined as localized collections of necrotic purulent material, often with infection. Reports of successful non-surgical treatment in dogs are scarce, but minimally invasive approaches are being explored. This case describes a five-month-old mixed-breed female dog with a two-week history of polyuria, polydipsia, and vomiting. Clinical examination revealed poor body condition and clinicopathological abnormalities included neutrophilic leukocytosis with a regenerative left shift, fasting hyperglycemia, elevated fructosamine, glycated hemoglobin, and β-hydroxybutyrate concentrations, with no evidence of pancreatitis or exocrine pancreatic insufficiency. Abdominal ultrasonography identified a thick-walled pancreatic cavity with mixed echogenic fluid. Ultrasound-guided drainage was safely performed, and cytology confirmed a pancreatic abscess with pyogranulomatous inflammation; cultures were negative. The dog received antibiotics and insulin, showing no recurrence on follow-up ultrasounds up to five months. One year post-discharge, the diabetes remained well managed. This case demonstrates successful treatment of a pancreatic abscess in a dog with juvenile diabetes mellitus using percutaneous drainage and medical management.

## 1. Introduction

Pancreatic abscesses are rarely documented in the veterinary literature. They are characterized as circumscribed collections of necrotic purulent material, potentially accompanied by infection, and are considered a possible complication of acute inflammatory pancreatopathy [1,2,3,4].

Several studies have documented a correlation between pancreatic abscesses and severe necrotizing pancreatitis or chronic-active pancreatitis [2,3,5,6,7,8]. Abscess formation is typically accompanied by necrosis, while anorexia, vomiting, and lethargy are the most commonly observed clinical signs of severe pancreatitis [3,5,7,8,9]. Abdominal ultrasound alone is often insufficient for definitive differentiation. However, the presence of compatible clinicopathologic findings is highly indicative of abscesses and is fundamental in distinguishing them from pancreatic pseudocysts, cystic neoplasms, or pancreatic phlegmons [10,11,12].

In human medicine, distinguishing between septic and sterile pancreatic fluid accumulations is crucial, as septic cases typically require surgical intervention, whereas sterile ones can be managed medically [13]. Less invasive techniques, such as percutaneous drainage catheter placement, have been associated with a lower incidence of new-onset multiple organ failure when compared to open necrosectomy, with minimal risk of further intervention, even in cases of infection [14,15,16,17]. A review of the veterinary literature reveals limited data regarding the effectiveness of conservative approaches for the management of pancreatic abscesses [3,18]. Despite a high case fatality rate in dogs, ranging from 33% to 86%, surgical intervention is often recommended, particularly in cases involving a septic process [2,3,5,8,18,19]. A feline case of an infected pancreatic abscess and concurrent diabetes mellitus (DM) was managed successfully with medical therapy and percutaneous drainage with ultrasound guidance on two occasions two weeks apart resulting in complete resolution of the abscess [20]. Successful medical management of pancreatic abscesses in dogs is not clearly documented in the veterinary literature; however, a recent retrospective study showed that it was a viable treatment option for some patients, and fine-needle aspiration appeared to be a safe minimally invasive procedure when performed under ultrasound guidance [18]. Previous veterinary reports concerning pancreatic pseudocysts similarly found no adverse events, such as leakage of pseudocyst contents [4]. Percutaneous drainage provided not only confirmation of an abscessation through cytological analysis and culture of the aspirated fluid but is also considered an effective alternative to surgical drainage for abscesses and cysts in organs like the pancreas [18,20,21]. However, potential risks associated with the procedure if not performed with proper expertise, include peritoneal contamination, hemorrhage, and secondary infection. Careful assessment of these risks is crucial when considering percutaneous drainage of abdominal abscesses [20].

DM is an uncommon disease in dogs under one year of age [22,23]. The majority of cases reported involve insulin-dependent dogs with various histopathologic abnormalities of the pancreas, but the etiology remains unclear [24]. This case report describes the clinical presentation and diagnostic approach in a young dog with juvenile DM, complicated by a pancreatic abscess. The aim of this article is to highlight the successful outcome of medical therapy in conjunction with percutaneous ultrasound-guided drainage of a pancreatic abscess in a juvenile diabetic dog.

## 2. Case Description

A five-month-old intact female mixed-breed dog weighing 2 kg was referred to the Clinic of Companion Animal Medicine, Aristotle University of Thessaloniki, Greece due to uncontrolled DM. The dog had a two-week history of polyuria, polydipsia, and vomiting and was diagnosed with DM by the referring veterinarian 5 days prior. Subcutaneous administration of 0.5 units per kg of porcine insulin zinc suspension (Caninsulin^®^, 40 IU/mL; MSD Animal Health, Boxmeer, Netherlands) every 12 h failed to effectively control the hyperglycemia. The dog lived outdoors, was unvaccinated, and had not received any antiparasitic treatment. On presentation, the dog was depressed and exhibited poor body condition (body condition score: 1.5/5) and growth retardation. A thorough clinical examination revealed pale oral mucous membranes, a weak pulse, prolonged capillary refill time (CRT > 2 s), and pale yellow ocular discharge. Ophthalmological examination identified keratoconjunctivitis sicca and cataract bilaterally. A complete blood count revealed mild anemia (Hct: 30.2%; reference interval [RI] 37–55%), marked leukocytosis (white blood cell count 50.2 K/μL; RI 6–17 K/μL), and neutrophilia (neutrophil count 40.5 K/μL; RI 3.9–8 K/μL) with a left shift. Serum biochemical analysis revealed fasting hyperglycemia (406 mg/dL; RI: 71–125 mg/dL), increased activity of serum ALT (85 U/L; RI 16–56 U/L) and γ-Gt (7 U/L; RI 1–3 U/L), hypocalcemia (5.2 mg/dL; RI 9.3–11. mg/dL 5), decreased ionized calcium levels (0.4 mg/dL; RI 0.6–1.3 mg/dL), and hyponatremia (136 mEq/L; RI 145–159 mEq/L). Additionally, serum fructosamine (336 µmol/L; RI 177–314 µmol/L), glycated hemoglobin A1C (4.3%; RI 0–3.1%), and b-hydroxybutyrate acid (5.9 mmol/L; RI < 0.6 mmol/L) were elevated, while other biochemical analyses and the acid–base balance remained within normal limits. The serum canine pancreatic lipase immunoreactivity (Spec cPL; IDEXX Laboratories, Inc., Kornwestheim, Germany) concentration (139 ug/L; RI < 200 μg/L not suspicious of pancreatitis) was within normal limits, while the serum canine trypsin-like immunoreactivity (cTLI; IDEXX Laboratories, Inc., Kornwestheim, Germany) (49.9 μg/L; RI 8.5–35 μg/L) was slightly elevated. Urinalysis revealed glycosuria and ketonuria.

**Figure 1:**
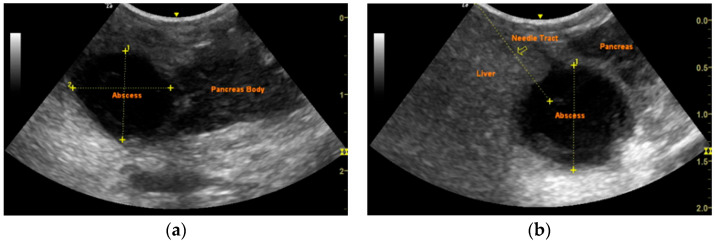
(**a**) Ultrasonographic appearance of the pancreatic abscess at the time of diagnosis: hypoechoic, oval cystic structure (2 × 1.2 × 1 cm) in the pancreatic body, with thick wall, containing mixed echogenic fluid in the pancreatic body; (**b**) the 22-gauge needle tract (yellow arrow) inserts into the abscess, after a short passage through liver parenchyma. Adjacent liver covers the insertion point after needle removal.

Supportive care included intravenous fluid therapy, intravenous administration of ampicillin–sulbactam (22 mg/kg q8h) (Begalin; Pfizer Hellas A.E., Neo Psychiko, Greece), marbofloxacin (5 mg/kg q24h) (Marbocyl^®^; Vetoquinol S.A., Lure, France), as well as a constant rate infusion of human regular insulin (Humulin^®^ Regular, 100 IU/mL; Eli Lilly and Company, Indianapolis, IN, USA). Abdominal ultrasonography revealed an oval cystic structure (2 × 1.2 × 1 cm), with a thick wall and mixed echogenic material in the pancreatic body (Figure 1a). Additional findings included hepatomegaly with diffuse hyperechogenicity and gallbladder lithiasis with small amount of biliary sludge.

**Figure 2:**
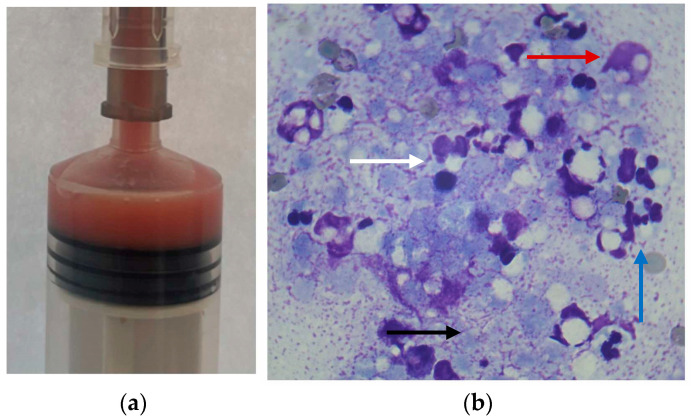
(**a**) A total of 2 ml of pink–white purulent fluid was aspirated using a 22-gauge needle from the pancreatic abscess; (**b**) cytological examination of the Giemsa-stained aspirate revealed degenerate (white arrow) and apoptotic (blue arrow) neutrophils, activated macrophages (red arrow), and necrotic debris (black), original magnification ×100.

The risks of surgical management for a suspected pancreatic abscessation in a dog with uncontrolled juvenile diabetes were taken into consideration, and conservative treatment was selected as the primary approach. Supportive care and antibiotic therapy were combined with ultrasound-guided drainage of the pancreatic fluid accumulation, which was performed under general anesthesia, after 48 h of hospitalization. A 22-gauge needle was inserted into the abscess after a short passage through the liver parenchyma. This approach allowed the adjacent liver tissue to cover the insertion site upon needle removal (Figure 1b). The needle was connected to a 25 cm three-way extension set to minimize movement during aspiration. A total of 2 mL of pink–white fluid (Figure 2a) was aspirated without immediate complications. Cytological examination of the Giemsa-stained aspirate indicated pyogranulomatous inflammation, consisting predominantly of degenerate neutrophils and occasional active macrophages. No microorganisms were observed (Figure 2b). Bacterial culture of the aspirated fluid was negative for both aerobic and anaerobic bacteria.

**Figure 3:**
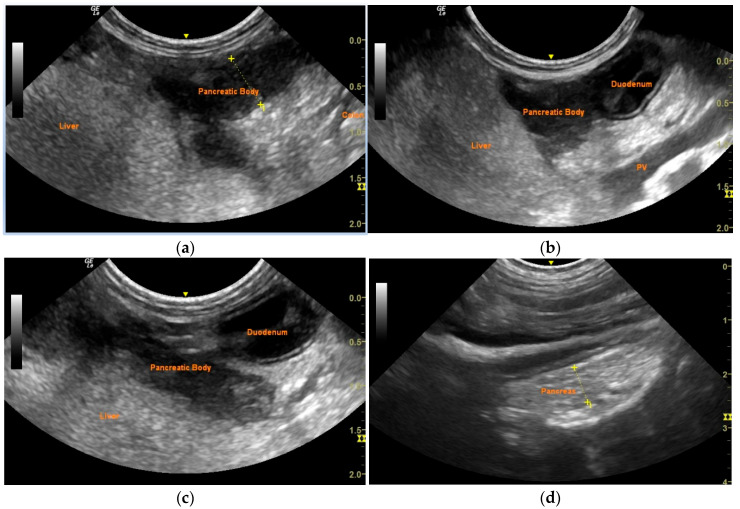
Follow-up ultrasonographic evaluations of the pancreas at 7 (**a**), 14 (**b**), and 21 (**c**) days and 5 months (**d**) after drainage of the pancreatic abscess. No signs of recurrence are observed.

Following resolution of laboratory abnormalities, the dog was discharged with subcutaneous administration of 1.5 units/kg of porcine lente insulin (Caninsulin^®^, 40 IU/mL; MSD Animal Health, Boxmeer, Netherlands) every 12 h. Antibiotic therapy was administered for a duration of two weeks. Follow-up ultrasonographic evaluations at 7, 14, and 21 days and 5 months post-drainage confirmed the absence of recurrence of the pancreatic abscess (Figure 3). DM was effectively managed, with stable control maintained for one year post-discharge.

## 3. Discussion

This case report describes the therapeutic approach for a dog with juvenile DM complicated by a pancreatic abscess. The primary clinical challenge was to establish a correlation between these conditions. An extensive investigation was conducted to determine whether the pancreatic abscess formation developed secondary to another underlying pancreatopathy. Additionally, it was uncertain whether DM was a pre-existing condition that was complicated by abscess-induced insulin resistance and resulted in ketosis, or whether the pancreatic abscess was contributing to the onset of DM.

A review of the veterinary literature identified five studies describing 73 cases of canine pancreatic abscesses, three of which also presented concurrent DM [2,3,5,6,7,8]. Pancreatic abscesses are defined as septic collections of purulent exudate and necrotic tissue within the pancreatic parenchyma and surrounding anatomical structures [1,3,25,26]. These lesions typically arise as a sequela of severe pancreatitis, leading to extensive parenchymal necrosis and are recognized in both human and veterinary medicine as late-stage complications, often associated with considerable morbidity [2,3,5,6,7,8,27,28,29]. Moreover, case reports have documented the coexistence of juvenile DM and exocrine pancreatic insufficiency (EPI) in canine patients [30,31,32]. In the present case, normal Spec cPL levels did not support pancreatitis as an underlying cause. Furthermore, in addition to the absence of overt clinical signs suggestive of EPI, the measurement of cTLI also excluded its presence. Therefore, insufficient evidence was present to support that an underlying pancreatitis was the primary etiological factor in pancreatic abscess formation, although pancreatic biopsies would be required to rule out pancreatitis.

Canine pancreatic abscesses are typically described as sterile, whereas microbial agents are isolated from most human pancreatic abscesses [2,3,6,7,8,18,28]. The prevalence of infection varies considerably between species. The reported positive culture rates for canine cases range from 15% to 25%, compared to rates of 40% to 70% in humans [7,8,18,33]. According to a recent retrospective study, bacterial culture was performed in 11 out of 15 canine cases, yielding positive results in four of these cases [18]. Two of these positive results were identified through cytological examination. Notably, a significant proportion of these dogs (7/11) had received antimicrobial therapy prior to sample collection, a factor that may have influenced the low yield of both aerobic and anaerobic cultures [18]. Pancreatic abscesses are typically polymicrobial infections involving both aerobic and anaerobic bacteria. *Escherichia coli* and *Staphylococcus pseudointermedius* have been previously reported in the veterinary literature as commonly isolated organisms [18]. Similarly, in the present case, bacterial culture was negative, and no microorganisms were identified on cytological evaluation, which may be a consequence of prior antibiotic treatment, although of a short duration. Broad-spectrum antibiotics such as marbofloxacin and ampicillin-sulbactam were administered, as they offer effective coverage against both aerobic and anaerobic pathogens.

In human medicine, open abdominal surgery has traditionally been performed after the patient is stabilized medically to manage pancreatic abscesses and confirm the diagnosis [27]. The emphasis on early surgical approaches, though, has decreased due to advances in non-invasive imaging, therapeutic techniques, and intensive care. It has been recently recommended that the initial management of pancreatic abscesses should prioritize percutaneous, endoscopic, and surgical drainage, combined with aggressive antimicrobial therapy [15,17,28]. Consequently, open surgery, minimally invasive surgery, endoscopic surgery, or a combination of these, are only suggested when minimally invasive approaches, such as percutaneous drainage, are ineffective in resolving a septic process [34]. In veterinary medicine, no universally accepted guidelines exist for the management of necrotic pancreatic masses, although surgical intervention remains a common approach [8,18,35]. Surgical drainage may be beneficial for dogs and cats with pancreatic abscesses; however, the risks and costs associated with this procedure often outweigh its potential benefits [2]. Based on the success of minimally invasive techniques in human medicine, it may be beneficial to investigate similar strategies in the veterinary field. A recent retrospective study involving 15 dogs diagnosed with pancreatic fluid accumulations, further supported the potential of minimally invasive approaches by reporting that ultrasound-guided aspiration was successful in all patients without complications [18]. Furthermore, surgical intervention resulted in favorable clinical outcomes in four of seven dogs, while medical management alone was successful in four of eight cases without the need for further intervention [18]. A larger study, which included 36 dogs who underwent surgery, reported a fair to grave prognosis, with 14 dogs euthanized and eight dying postoperatively [8]. The high mortality rates associated with surgery in both fields have led to a shift towards conservative management as a preferred approach or precursor to more invasive procedures [4,18,35]. This information highlights the potential benefits of a conservative approach and minimally invasive strategies at least initially, in order to achieve optimal outcomes in veterinary patients with pancreatic abscesses or necrotic collections.

In this case report, the patient was deemed unstable for surgical intervention due to concurrent ketosis. As the safety of ultrasound-guided fine-needle aspiration of pancreatic fluid accumulations has been established, medical management, proven effective in some dogs, was chosen as the initial treatment option. Conservative treatment, including ketoacidosis management, antibiotic therapy, and percutaneous ultrasound-guided drainage of the pancreatic abscess, provided a viable alternative. The prognosis was excellent, with no need for further interventions and sustained glycemic control after a single drainage procedure.

## 4. Conclusions

To the best of the authors’ knowledge, this is the first report of a pancreatic abscess in a dog with juvenile DM successfully managed with a combination of medical treatment and a single percutaneous ultrasound-guided drainage. This case demonstrates the potential effectiveness of less invasive techniques for treating pancreatic abscesses in critically ill patients. Further investigation is necessary to assess the long-term outcomes of this treatment approach.

## Data Availability

No new data were created or analyzed in this case report. Data sharing is not applicable to this article.

## Abbreviations

The following abbreviations are used in this manuscript:

DM	Diabetes mellitus
CRT	Capillary refill time
Spec cPL	Specific pancreatic lipase
cTLI	Canine trypsin-like immunoreactivity
EPI	Exocrine pancreatic insufficiency

## References

[1] Warshaw A.L. (1974). Inflammatory Masses Following Acute Pancreatitis: Phlegmon, Pseudocyst, and Abscess. Surg. Clin. N. Am..

[2] Salisbury S.K., Lantz G.C., Nelson R.W., Kazacos E.A. (1988). Pancreatic Abscess in Dogs: Six Cases (1978–1986). J. Am. Vet. Med. Assoc..

[3] Edwards D., Bauer M., Walker M., Pardo A.D., McCracken M., Walker T. (1990). Pancreatic Masses in Seven Dogs Following Acute Pancreatitis. J. Am. Anim. Hosp. Assoc..

[4] VanEnkevort B.A., O’Brien R.T., Young K.M. (1999). Pancreatic Pseudocysts in 4 Dogs and 2 Cats: Ultrasonographic and Clinicopathologic Findings. J. Vet. Intern. Med..

[5] Stimson E.L., Espada Y., Moon M., Troy G.C. (1998). Pancreatic Abscess in Nine Dogs. J. Vet. Intern. Med..

[6] Coleman M., Robson M. (2005). Pancreatic Masses Following Pancreatitis: Pancreatic Pseudocysts, Necrosis, and Abscesses. Contin. Educ. Vet..

[7] Johnson M.D., Mann F.A. (2006). Treatment for Pancreatic Abscesses via Omentalization with Abdominal Closure versus Open Peritoneal Drainage in Dogs: 15 Cases (1994–2004). J. Am. Vet. Med. Assoc..

[8] Anderson J.R., Cornell K.K., Parnell N.K., Salisbury S.K. (2008). Pancreatic Abscess in 36 Dogs: A Retrospective Analysis of Prognostic Indicators. J. Am. Anim. Hosp. Assoc..

[9] Akol K.G., Washabau R.J., Saunders H.M., Hendrick M.J. (1993). Acute Pancreatitis in Cats with Hepatic Lipidosis. J. Vet. Intern. Med..

[10] Saunders H.M., VanWinkle T.J., Drobatz K., Kimmel S.E., Washabau R.J. (2002). Ultrasonographic Findings in Cats with Clinical, Gross Pathologic, and Histologic Evidence of Acute Pancreatic Necrosis: 20 Cases (1994–2001). J. Am. Vet. Med. Assoc..

[11] Hecht S., Henry G. (2007). Sonographic Evaluation of the Normal and Abnormal Pancreas. Clin. Tech. Small Anim. Pract..

[12] Rademacher N., Ohlerth S., Scharf G., Laluhova D., Sieber-Ruckstuhl N., Alt M., Roos M., Grest P., Kaser-Hotz B. (2008). Contrast-Enhanced Power and Color Doppler Ultrasonography of the Pancreas in Healthy and Diseased Cats. J. Vet. Intern. Med..

[13] Bradley E.L. (1993). A clinically based classification system for acute pancreatitis. Summary of the international symposium on acute pancreatitis, Atlanta, Ga, September 11 through 12, 1992. Arch. Surg..

[14] van Santvoort H.C., Besselink M.G., Bakker O.J., Hofker H.S., Boermeester M.A., Dejong C.H., van Goor H., Schaapherder A.F., van Eijck C.H., Bollen T.L. (2010). A Step-up Approach or Open Necrosectomy for Necrotizing Pancreatitis. N. Engl. J. Med..

[15] Varadarajulu S., Bang J.H., Phadnis M.A., Christein J.D., Wilcox C.M. (2011). Endoscopic Transmural Drainage of Peripancreatic Fluid Collections: Outcomes and Predictors of Treatment Success in 211 Consecutive Patients. J. Gastrointest. Surg..

[16] Hollemans R.A., Bakker O.J., Boermeester M.A., Bollen T.L., Bosscha K., Bruno M.J., Buskens E., Dejong C.H., van Duijvendijk P., van Eijck C.H. (2019). Superiority of Step-up Approach vs Open Necrosectomy in Long-Term Follow-up of Patients with Necrotizing Pancreatitis. Gastroenterology.

[17] Goyal J., Ramesh J. (2014). Endoscopic Management of Peripancreatic Fluid Collections. Frontline Gastroenterol..

[18] Talbot C.T., Cheung R., Holmes E.J., Cook S.D. (2022). Medical and Surgical Management of Pancreatic Fluid Accumulations in Dogs: A Retrospective Study of 15 Cases. J. Vet. Intern. Med..

[19] Nemoto Y., Haraguchi T., Shimokawa Miyama T., Kobayashi K., Hama K., Kurogouchi Y., Fujiki N., Baba K., Okuda M., Mizuno T. (2017). Pancreatic Abscess in a Cat Due to *Staphylococcus aureus* Infection. J. Vet. Med. Sci..

[20] Lee M., Kang J.-H., Chang D., Na K.-J., Yang M.-P. (2015). Pancreatic Abscess in a Cat with Diabetes Mellitus. J. Am. Anim. Hosp. Assoc..

[21] Smith S., Biller D. (1998). Resolution of a Pancreatic Pseudocyst in a Dog Following Percutaneous Ultrasonographic-Guided Drainage. J. Am. Anim. Hosp. Assoc..

[22] Greco D.S., Chastain C.B., Hoskins J.D. (2001). Endocrine and Metabolic System. Veterinary Pediatrics: Dogs and Cats from Birth to Six Months.

[23] Greco D.S. (2006). Pediatric Endocrinology. Vet. Clin. Small Anim. Pract..

[24] Gilor C., Niessen S.J.M., Furrow E., DiBartola S.P. (2016). What’s in a Name? Classification of Diabetes Mellitus in Veterinary Medicine and Why It Matters. J. Vet. Intern. Med..

[25] Warshaw A.L., Richter J.M. (1984). A Practical Guide to Pancreatitis. Curr. Probl. Surg..

[26] Warshaw A.L., Jin G.L. (1985). Improved Survival in 45 Patients with Pancreatic Abscess. Ann. Surg..

[27] Saxon A., Reynolds J.T., Doolas A. (1981). Management of Pancreatic Abscesses. Ann. Surg..

[28] Amano H., Takada T., Isaji S., Takeyama Y., Hirata K., Yoshida M., Mayumi T., Yamanouchi E., Gabata T., Kadoya M. (2009). Therapeutic Intervention and Surgery of Acute Pancreatitis. J. Hepatobiliary Pancreat. Sci..

[29] Schaer M., Washabau R.J., Day M.J. (2013). Abscess, Necrosis, Pseudocyst, Phlegmon, and Infection. Canine & Feline Gastroenterology.

[30] Kang J.-H., Na K.-J., Mo I.-P., Chang D., Yang M.-P. (2008). Juvenile Diabetes Mellitus Accompanied by Exocrine Pancreatic Insufficiency in a Dog. J. Vet. Med. Sci..

[31] Mamom T., Rungpupradit J. (2010). Diabetes Mellitus Concurrent with Exocrine Pancreatic Insufficiency in a Young Golden Retriever Dog: A Clinicopathological Report. J. Mahanakorn Vet. Med..

[32] Neiger R., Jaunin V.B., Boujon C.E. (1996). Exocrine Pancreatic Insufficiency Combined with Insulin-Dependent Diabetes Mellitus in a Juvenile German Shepherd Dog. J. Small Anim. Pract..

[33] Chen J., Fukami N., Li Z. (2012). Endoscopic Approach to Pancreatic Pseudocyst, Abscess and Necrosis: Review on Recent Progress. Dig. Endosc..

[34] Leppäniemi A., Tolonen M., Tarasconi A., Segovia-Lohse H., Gamberini E., Kirkpatrick A.W., Ball C.G., Parry N., Sartelli M., Wolbrink D. (2019). 2019 WSES Guidelines for the Management of Severe Acute Pancreatitis. World J. Emerg. Surg..

[35] Thompson L.J., Seshadri R., Raffe M.R. (2009). Characteristics and Outcomes in Surgical Management of Severe Acute Pancreatitis: 37 Dogs (2001–2007). J. Vet. Emerg. Crit. Care.

